# Supplementary material for: Development of a CRISPR/Cas9-induced gene editing system for Pseudoalteromonas fuliginea and its applications in functional genomics
Source: Appl Environ Microbiol. 2025 Oct 29;91(11):e01771-25. doi: 10.1128/aem.01771-25 (PMC12628799; doi:10.1128/aem.01771-25)
Supplement: Supplemental figures — Figures S1 to S7. [file aem.01771-25-s0002.docx]

**Supplementary Figures**

**Development of a CRISPR/Cas9-induced gene editing system for *Pseudoalteromonas fuliginea* and its applications in functional genomics**

Zedong Duan^1,2^, Ruyi Yang^1,3^, Tingyi Lai^1,2^, Wanning Jiang^1,2^, Jin Zhang^1^, Bo Chen^1^, Li Liao^1,2#^

^1^ Key Laboratory for Polar Science; Arctic Yellow River Earth System National Observation and Research Station, Ministry of Natural Resources, Polar Research Institute of China, Shanghai, China.

^2^ Key Laboratory of Polar Ecosystem and Climate Change, Ministry of Education; Shanghai Key Laboratory of Polar Life and Environment Sciences; and School of Oceanography, Shanghai Jiao Tong University, Shanghai, 200030, China.

^3^ School of Health Science and Engineering, University of Shanghai for Science and Technology, Shanghai, 200093, China.

^#^ Address correspondence to Li Liao. liaoli@pric.org.cn.


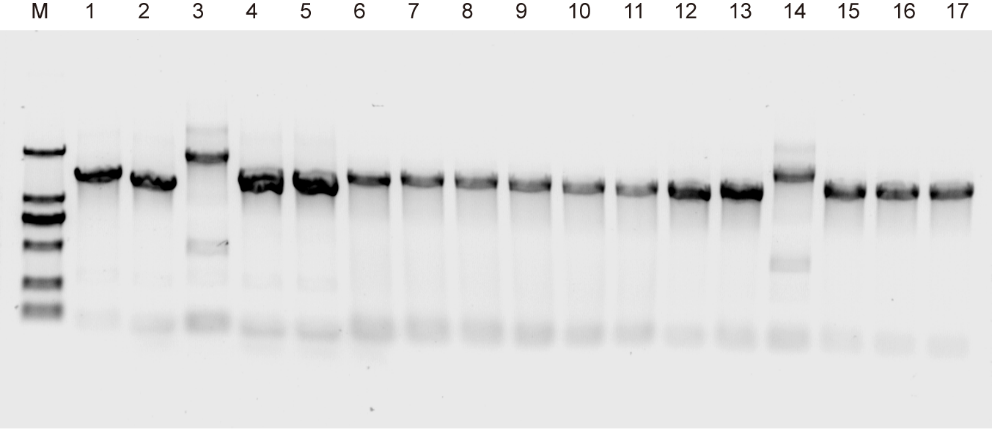


**Figure S1**. The gene editing efficiency of *fliJ* in *P. fuliginea* BSW20308 was determined based on the PCR and Gel Electrophoresis results.

**
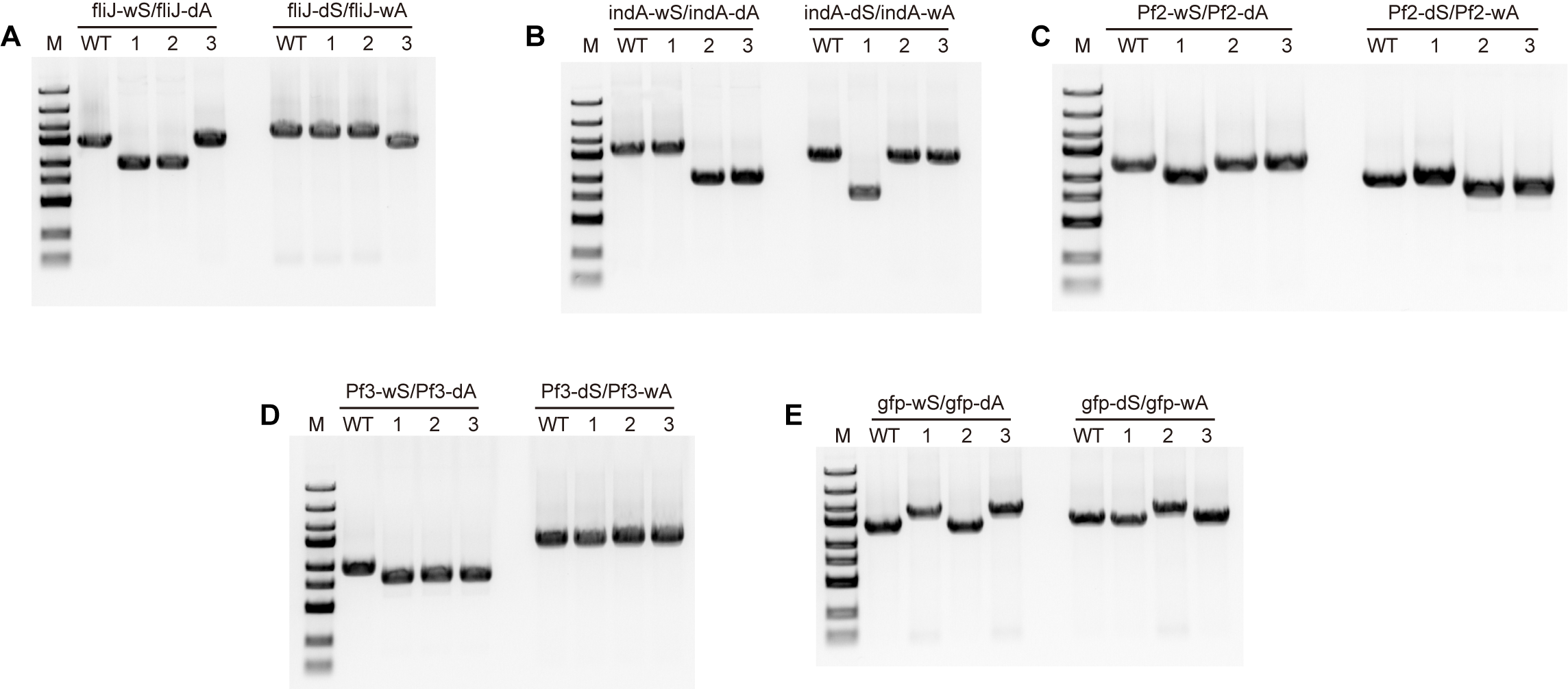
**

**Figure S2**. PCR detection of single crossover mutants using two primer pairs. (**A**) *fliJ* locus. (**B**) *indA* locus. (**C**) *Pf2* locus. (**D**) *Pf3* locus. (**E**) *gfp* insertion. Lane M: DNA marker; WT: wild-type control; lanes 1–3: independent colonies tested.


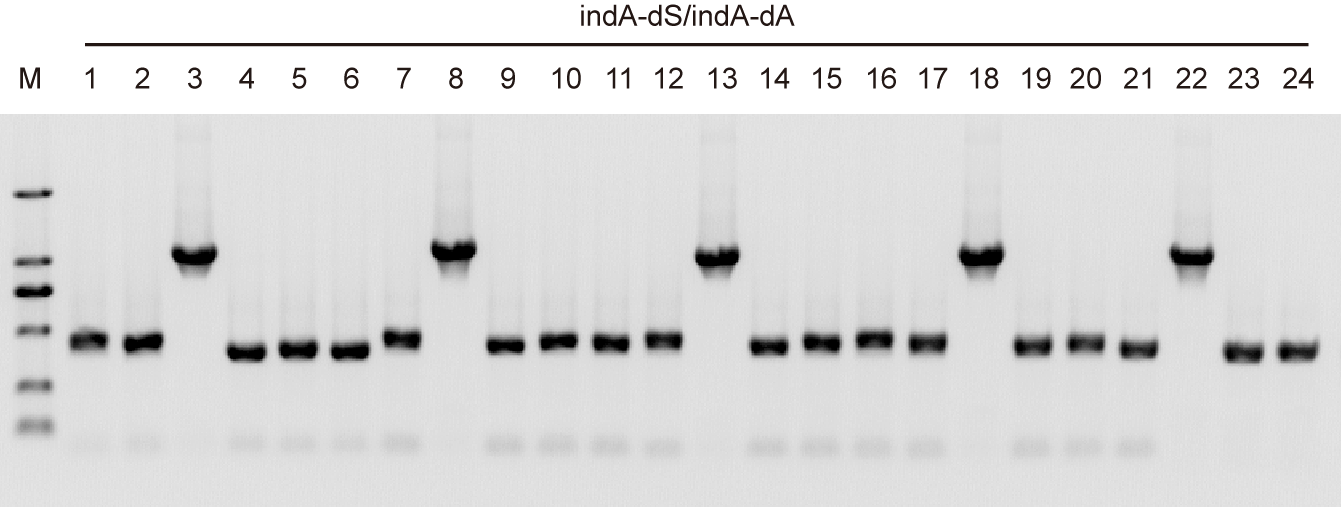


**Figure S3**. The gene editing efficiency of *indA* gene in *P. fuliginea* BSW20308 was determined based on the PCR and Gel Electrophoresis results.


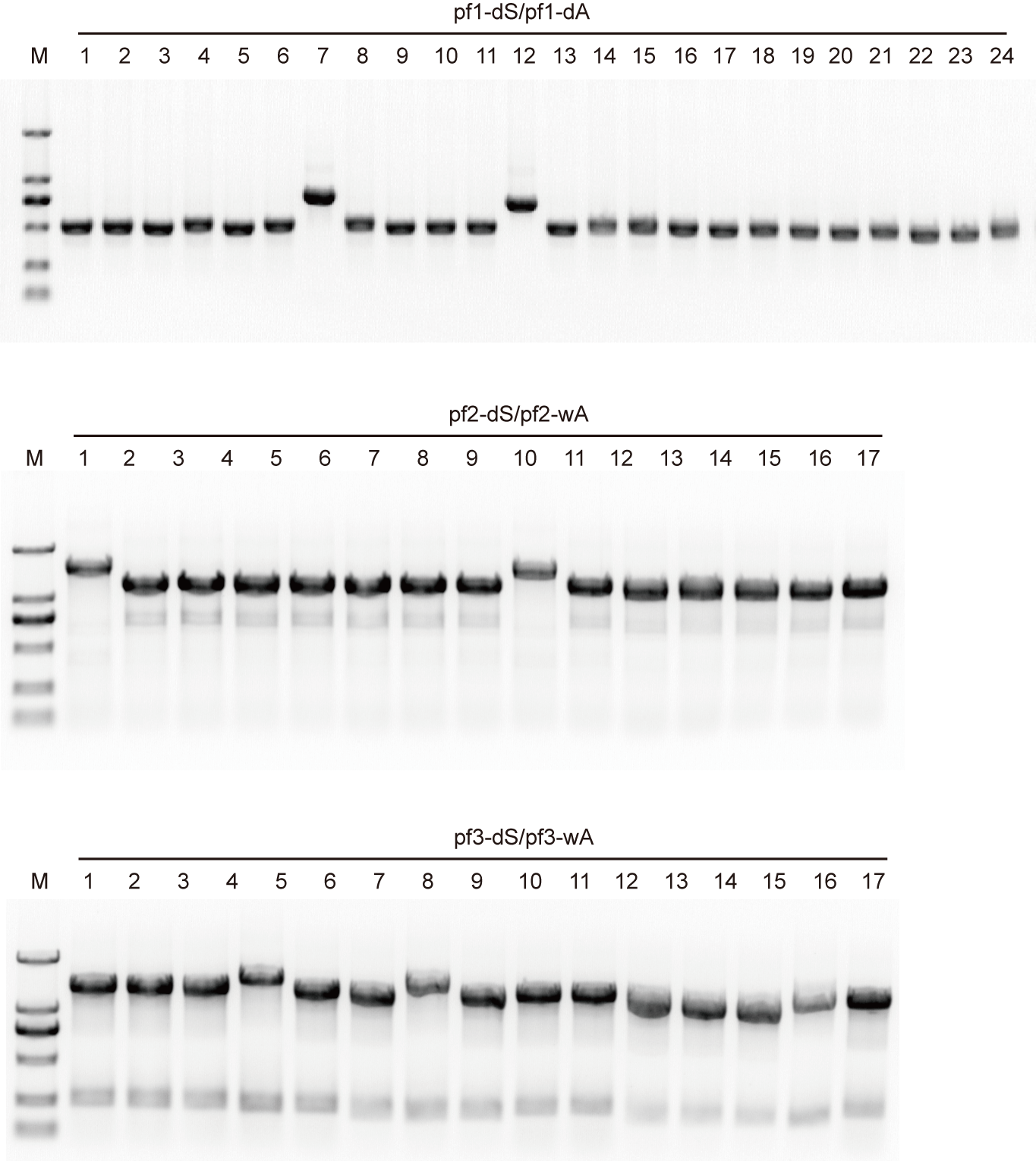


**Figure S4**. The gene editing efficiency of Pf1, Pf2 and Pf3 in *P. fuliginea* BSW20308 was determined based on the PCR and Gel Electrophoresis results.


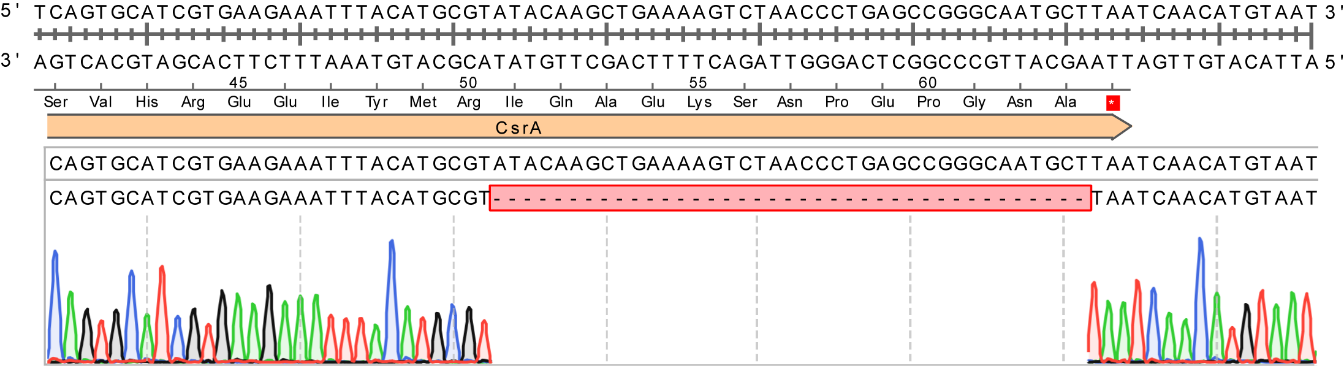


**Figure S5. C‑terminal truncation of *csrA* in *P. fuliginea* via CRISPR/Cas9**. Sanger sequencing of PCR products confirmed a 39‑bp deletion at the 3′ end of *csrA*, resulting in a C‑terminal truncation (predicted protein length 50 aa).


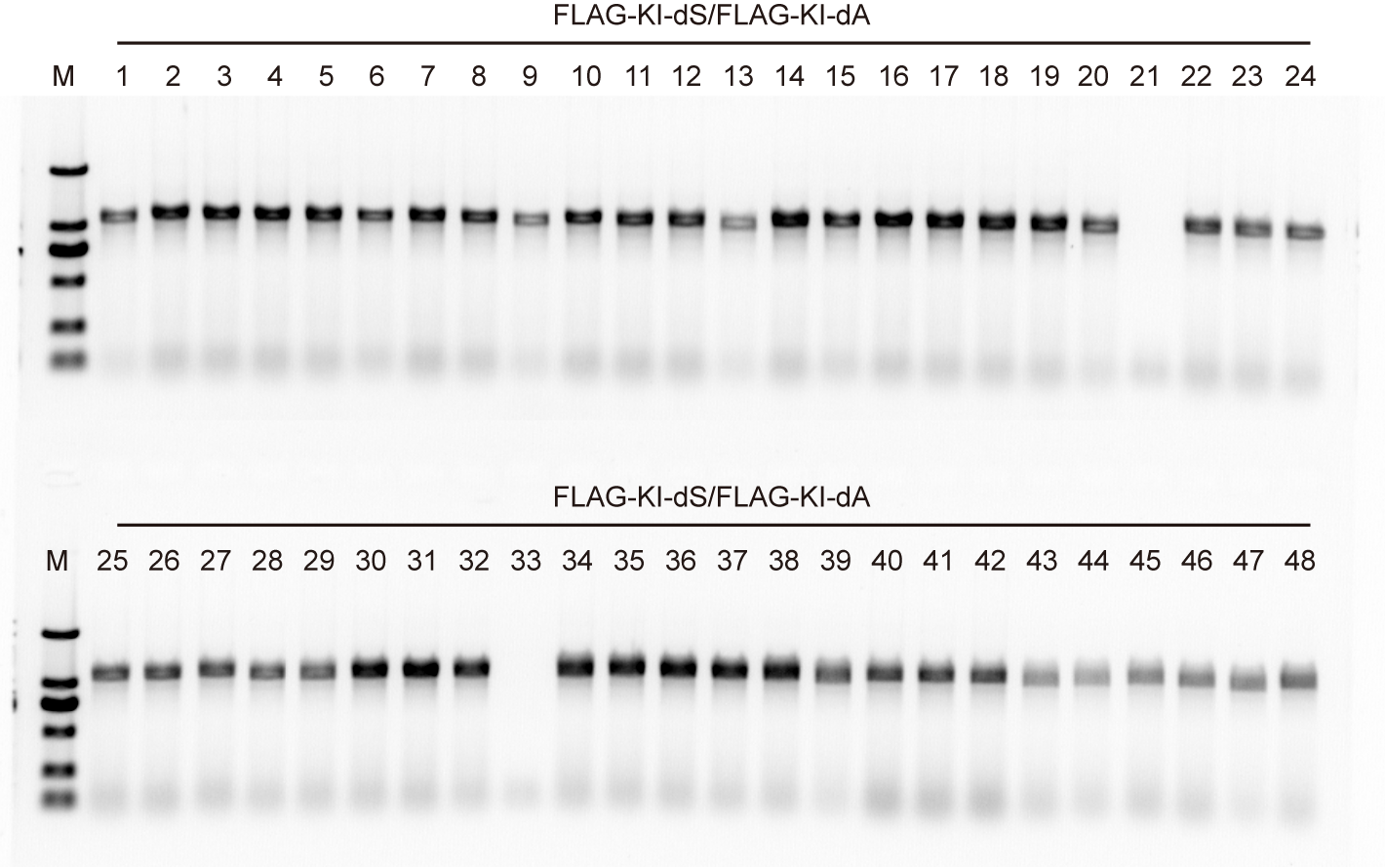


**Figure S6**. The gene editing efficiency of 3×FLAG tag in *P. fuliginea* BSW20308 was determined based on the PCR and Gel Electrophoresis results.


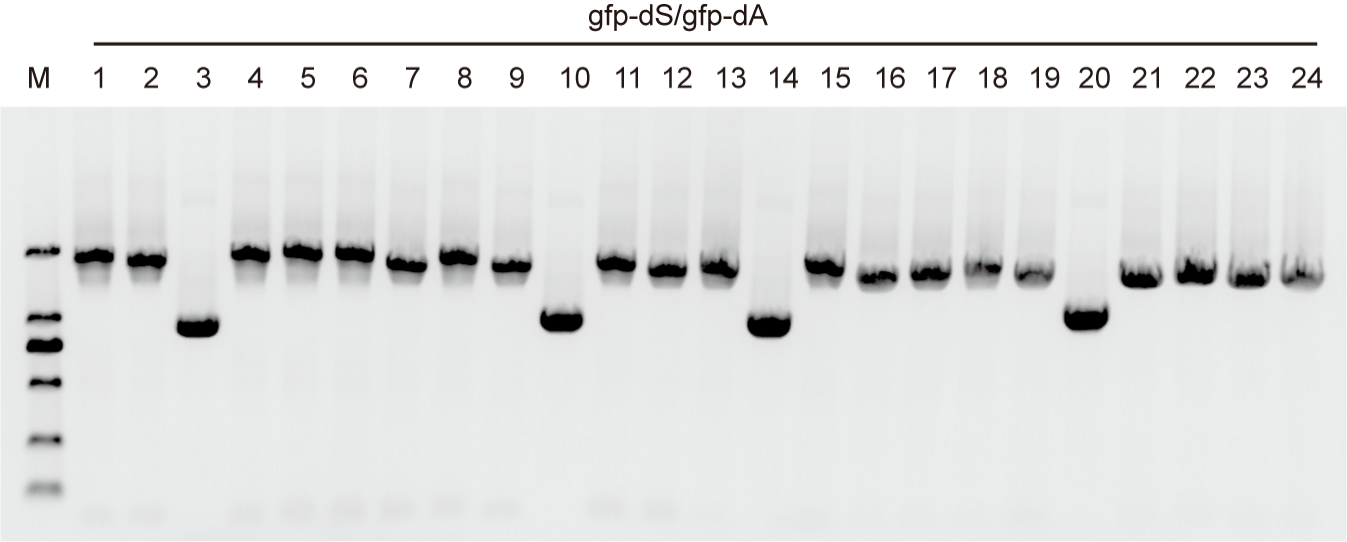


**Figure S7**. The gene inserting efficiency of *gfp* gene in *P. fuliginea* BSW20308 was determined based on the PCR and gel electrophoresis results.
